# Supplementary material for: Exploring the Validity of the 14-Item Mediterranean Diet Adherence Screener (MEDAS): A Cross-National Study in Seven European Countries around the Mediterranean Region
Source: Nutrients. 2020 Sep 27;12(10):2960. doi: 10.3390/nu12102960 (PMC7601687; doi:10.3390/nu12102960)
Supplement: Supplementary file 1 [file nutrients-12-02960-s001.zip › Table S9.docx]

**Supplementary Table S9.-** Agreement between the FFQ-MEDAS and the 3d-FD: per-item validation analysis (κappa statistics) in the sample population from Spain.

| Question | Score | 3d-FD  (% scoring 1) | FFQ-MEDAS^1^  (% scoring 1) | % Absolute agreement | κ (95%CI)  (3d-FD *vs* FFQ-MEDAS(1) | κ (95%CI)  (3d-FD *vs* FFQ-MEDAS(2) | κ (mean)  Level of agreement^4^ |
| --- | --- | --- | --- | --- | --- | --- | --- |
| 1.- Olive oil | yes | 82.5 | 100.0 | 82.5 | NA^2^ | NA | NA |
| 2.- Olive oil | ≥4 | 7.5 | 38.8 | 68.8 | 0.238  (0.123, 0.599) | 0.217  (-0.133, 0.567) | 0.228  Fair |
| 3.- Vegetables | ≥2 | 40.0 | 62.5 | 47.5 | -0.058  (-0.354, 0.239) | 0.057  (-0.236, 0.349) | 0.000  No agreement |
| 4.- Fresh fruits | ≥3 | 27.5 | 36.3 | 76.3 | 0.480  (0.181, 0.779) | 0.437  (0.134, 0.739) | 0.459  Moderate |
| 5.- Red & processed meat | <1 | 82.5 | 95.0 | 77.5 | -0.117  (-0.717, 0.482) | -0.046  (-0.694, 0.602) | -0.08  No agreement |
| 6.- Butter, margarine | <1 | 97.5 | 95.0 | 97.5 | 0.655  (-0.012, 1.323) | 0.655  (-0.012, 1.323) | 0.655  Good |
| 7.- Sweet beverages | <1 | 95.0 | 92.5 | 92.5 | 0.362  (-0.333, 1.056) | 0.362  (-0.333, 1.056) | 0.362  Fair |
| 8.- Wine | 7 to14 | 12.5 | 5.0 | 92.5 | 0.538  (0.036, 1.041) | 0.538  (0.036, 1.041) | 0.538  Moderate |
| 9.- Legumes | ≥3 | 7.5 | 13.8 | 86.3 | 0.219  (-0.422, 0.859) | 0.330  (-0.165, 0.824) | 0.275  Fair |
| 10.- Fish & seafood | ≥3 | 52.5 | 22.5 | 67.5 | 0.366  (0.083, 0.649) | 0.366  (0.083, 0.649) | 0.366  Fair |
| 11.- Desserts | <3 | 80.0 | 71.3 | 81.3 | 0.474  (0.147, 0.800) | 0.521  (0.198, 0.843) | 0.498  Moderate |
| 12.- Nuts | ≥3 | 30.0 | 32.5 | 85.0 | 0.659  (0.408, 0.911) | 0.659  (0.408, 0.911) | 0.659  Good |
| 13.- White over red meat^3^ | ≤1 or yes | 32.5 | 87.5 | 40.0 | 0.076  (-0.170, 0.322) | 0.023  (-0.211, 0.258) | 0.050  Slight |
| 14.- ‘Sofrito’ | ≥2 | 20.0 | 77.5 | 32.5 | -0.063  (-0.292, 0.166) | 0.029  (-0.180, 0.238) | 0.029  Slight |
| Mean value |  | 47.7 | 59.3 | 73.4 |  |  |  |

^1^: Mean value of FFQ-MEDAS (1) and FFQ-MEDAS (2); ^2^: Not applicable (one of the variables is a constant when all answers scored the same value); ^3^: ≤1 for the 3d-FD and 'yes' for the FFQ-MEDAS; ^4^ к ≤ 0 no agreement (small negative values) or disagreement (large negative values), к = 0.01 − 0.20 slight, к = 0.21 − 0.40 fair, к = 0.41 − 0.60 moderate, к = 0.61 − 0.80 substantial, к = 0.81 – 1.0 almost perfect [26].
