# Supplementary material for: Exploring the Validity of the 14-Item Mediterranean Diet Adherence Screener (MEDAS): A Cross-National Study in Seven European Countries around the Mediterranean Region
Source: Nutrients. 2020 Sep 27;12(10):2960. doi: 10.3390/nu12102960 (PMC7601687; doi:10.3390/nu12102960)
Supplement: Supplementary file 1 [file nutrients-12-02960-s001.zip › Table S1.docx]

**Supplementary Table S1.-** Criteria to calculate the 14-MEDAS score from the designed food frequency questionnaire (FFQ- MEDAS) and from the 3-day food diaries (3d-FD).

| Questions from FFQ-MEDAS | Response options and scoring criteria to calculate MEDAS from FFQ-MEDAS | Scoring criteria to calculate MEDAS from 3d-FD |
| --- | --- | --- |
| 1.Do you use olive oil as your main culinary fat? | Yes = 1  No = 0 | For each day: Yes=1; No=0; add day1+day2+day3; if sum is >= 2 then score = 1, if sum is <2 then score = 0 |
| 2. How many tablespoons of olive oil do you consume per day? | Four or more = 1  One or less; two or three = 0 | Calculate mean number of tablespoons per day; if mean≥4, score=1; if mean< 4, score=0 |
| 3. How many vegetable servings do you consume per day? (Including cooked and raw vegetables; potatoes and beans are not included; one serving = one large cup or half a large plate) | Two; three or more = 1  Less than one; one; two = 0 | Calculate mean servings per day; if mean≥2, score =1; if mean<2 score=0 |
| 4. How many servings of fresh fruit do you consume per day? (One serving = one fruit unit of medium size, one large cup of sliced fruit, one slice of melon or watermelon of medium size, or one cup of freshly squeezed juice). | Three or more = 1  Less than one; one; two = 0 | Calculate mean servings per day; if mean≥3, score =1; if mean<3, score=0 |
| 5. How many servings of red meat, or red meat products do you consume per week? (Red meat: veal/beef, pork, lamb; derived products: hamburguers, sausages, ham, etc, you may include here some examples of meat products typical from your country. One serving = 100 to 150 g = a quarter to half a meal dish) | One or less; two to four; five to six = 1  Seven or more =0 (corresponds to one or more per day) | Calculate mean servings per day; if <1, score=1; if ≥1, score= 0 |
| 6. How many servings of butter, margarine, or cream do you consume per day? (One serving = 12 g = one dessert spoon of butter and margarine; 2 tablespoons for cream.) | Less than one = 1  One; more than one = 0 | Calculate mean servings per day; if <1, score=1; if ≥1, score= 0 |
| 7. How many sweet/fizzy beverages/sodas do you consume per day? (You may include some examples of juice fruits or soft drinks with added sugar commonly consumed in the country) | Less than one = 1  One; more than one = 0 | Calculate mean number per day; if <1, score=1; if ≥1, score= 0 |
| 8. How many glasses/cups of wine do you consume per week? | Seven to fourteen glasses (one or two glasses per day) =1  one or less (occasionally); two to six (sometimes but not daily); more than fourteen (more than two glasses per day) = 0 | Calculate mean glasses per day and estimate week portion; if number of glasses per week is seven to fourteen score=1; otherwise score=0 |
| 9. How many servings of legumes do you consume per week? (Including beans, peas, chickpeas, lentils, etc. One serving = 150 g = 1 plate or 1 cup.) | Three or more = 1  Less than one; one; two = 0 | Calculate mean servings per day and estimate week portion; if ≥3, score =1; if <3, score=0 |
| 10. How many servings of fish or shellfish do you consume per week? (One serving = 100 to 150 g = a quarter to half a meal dish) | Three or more = 1  Less than one; one; two = 0 | Calculate mean servings per day and estimate week portion; if ≥3, score =1; if <3, score=0 |
| 11. How many times per week do you consume industrial (not homemade) desserts/sweets/pastries? (Including cakes, cookies, biscuits, ice-creams, custard, etc) | Less than one; one; two = 1  Three; four or more = 0 | For each day code 1 if dessert/etc was eaten; code for 0 if no consumption; sum the three days; if sum ≤1, score=1; if sum >1, score=0 |
| 12. How many servings of (unsalted) nuts do you consume per week? (Including unsalted peanuts, almonds, hazelnuts, chestnut, walnuts, pecan nuts, etc. One serving = 30 g = one handful) | Three or more = 1  Less than one; one; two = 0 | Calculate mean servings per day and estimate week portion; if ≥3, score =1; if <3, score=0 |
| 13. Do you preferentially consume chicken, turkey or rabbit meat, or a vegetarian protein source, instead of red meat or any derived products? | Yes = 1 (1 point for vegetarians)  No = 0 | For each day code 1 if red meat was eaten; code 0 if no consumption; sum the three days; if sum ≤1, score=1; if sum >1, score=0 |
| 14. How many times per week do you consume dishes cooked with tomato or tomato sauce, onion and (or) garlic, and olive oil? | Two or more = 1  Less than one; one = 0 | For each meal cooked with tomato/etc score=1; otherwise score=0. Calculate mean of three days; if mean ≥2, score=1; if mean <2, score=0 |
