# Supplementary material for: Exploring the Validity of the 14-Item Mediterranean Diet Adherence Screener (MEDAS): A Cross-National Study in Seven European Countries around the Mediterranean Region
Source: Nutrients. 2020 Sep 27;12(10):2960. doi: 10.3390/nu12102960 (PMC7601687; doi:10.3390/nu12102960)
Supplement: Supplementary file 1 [file nutrients-12-02960-s001.zip › Table S2.docx]

**Supplementary Table S2.-** Agreement between the FFQ-MEDAS and the 3d-FD: per-item validation analysis (κappa statistics) in the whole sample population (all countries).

| Question | Score | 3d-FD  (% scoring 1) | FFQ-MEDAS^1^  (% scoring 1) | % Absolute agreement | κ (95%CI)  (3d-FD *vs* FFQ-MEDAS(1) | κ (95%CI)  (3d-FD *vs* FFQ-MEDAS(2) | κ (mean)  Level of agreement^3^ |
| --- | --- | --- | --- | --- | --- | --- | --- |
| 1.- Olive oil | yes | 62.0 | 76.3 | 82.1 | 0.602  (0.518, 0.685) | 0.578  (0.493, 0.664) | 0.590  Moderate |
| 2.- Olive oil | ≥4 | 15.0 | 19.1 | 81.8 | 0.336  (0.202, 0.469) | 0.385  (0.257, 0.513) | 0.361  Fair |
| 3.- Vegetables | ≥2 | 31.7 | 57.3 | 57.0 | 0.192  (0.101, 0.283) | 0.176  (0.085, 0.266) | 0.184  Slight |
| 4.- Fresh fruits | ≥3 | 18.2 | 21.1 | 84.3 | 0.479  (0.365, 0.592) | 0.525  (0.415, 0.634) | 0.502  Moderate |
| 5.- Red & processed meat | <1 | 66.3 | 70.0 | 61.4 | 0.110  (0.002, 0.219) | 0.117  (0.009, 0.224) | 0.114  Slight |
| 6.- Butter, margarine | <1 | 75.8 | 62.1 | 67.4 | 0.213  (0.111, 0.316) | 0.300  (0.197, 0.404) | 0.257  Fair |
| 7.- Sweet beverages | <1 | 81.6 | 66.4 | 71.5 | 0.299  (0.189, 0.409) | 0.263  (0.154, 0.373) | 0.281  Fair |
| 8.- Wine | 7 to14 | 10.9 | 7.1 | 89.5 | 0.343  (0.161, 0.524) | 0.439  (0.268, 0.609) | 0.391  Fair |
| 9.- Legumes | ≥3 | 18.0 | 17.7 | 78.4 | 0.238  (0.102, 0.374) | 0.289  (0.154, 0.423) | 0.264  Fair |
| 10.- Fish & seafood | ≥3 | 29.8 | 12.5 | 73.5 | 0.238  (0.116, 0.360) | 0.239  (0.116, 0.362) | 0.239  Fair |
| 11.- Desserts | <3 | 43.8 | 64.0 | 65.5 | 0.313  (0.224, 0.402) | 0.353  (0.265, 0.441) | 0.333  Fair |
| 12.- Nuts | ≥3 | 17.2 | 21.7 | 81.2 | 0.403  (0.284, 0.523) | 0.402  (0.218, 0.522) | 0.403  Fair |
| 13.- White over red meat^2^ | ≤1 or yes | 47.1 | 69.8 | 60.8 | 0.250  (0.158, 0.342) | 0.217  (0.125, 0.310) | 0.234  Fair |
| 14.- ‘Sofrito’ | ≥2 | 30.1 | 28.8 | 57.7 | 0.170  (0.080, 0.260) | 0.238  (0.149, 0.328) | 0.204  Slight |
| Mean value |  | 39.1 | 42.4 | 72.3 |  |  |  |

^1^: Mean value of FFQ-MEDAS (1) and FFQ-MEDAS (2); ^2^: ≤1 for the 3d-FD and 'yes' for the FFQ-MEDAS; ^3^ к ≤ 0 no agreement (small negative values) or disagreement (large negative values), к = 0.01 − 0.20 slight, к = 0.21 − 0.40 fair, к = 0.41 − 0.60 moderate, к = 0.61 − 0.80 substantial, к = 0.81 – 1.0 almost perfect [26].
