# Supplementary material for: Exploring the Validity of the 14-Item Mediterranean Diet Adherence Screener (MEDAS): A Cross-National Study in Seven European Countries around the Mediterranean Region
Source: Nutrients. 2020 Sep 27;12(10):2960. doi: 10.3390/nu12102960 (PMC7601687; doi:10.3390/nu12102960)
Supplement: Supplementary file 1 [file nutrients-12-02960-s001.zip › Table S3.docx]

**Supplementary Table S3.-** Agreement between the FFQ-MEDAS and the 3d-FD: per- item validation analysis (κappa statistics) in the sample population from Italy.

| Question | Score | 3d-FD  (% scoring 1) | FFQ-MEDAS^1^  (% scoring 1) | % Absolute agreement | κ (95%CI)  (3d-FD *vs* FFQ-MEDAS(1) | κ (95%CI)  (3d-FD *vs* FFQ-MEDAS(2) | κ (mean)  Level of agreement^4^ |
| --- | --- | --- | --- | --- | --- | --- | --- |
| 1.- Olive oil | yes | 100.0 | 98.3 | 98.3 | NA^2^ | NA | NA |
| 2.- Olive oil | ≥4 | 3.4 | 19.8 | 76.7 | -0.062  (-0.570, 0.447) | -0.063  (-0.548, 0.422) | -0.063  No agreement |
| 3.- Vegetables | ≥2 | 24.1 | 47.1 | 71.6 | 0.470  (0.228, 0.712) | 0.367  (0.134, 0.601) | 0.419  Moderate |
| 4.- Fresh fruits | ≥3 | 3.4 | 12.1 | 87.9 | 0.153  (-0.391, 0.698) | 0.209  (-0.390, 0.808) | 0.181  Slight |
| 5.- Red & processed meat | <1 | 65.5 | 95.7 | 68.1 | 0.127  (-0.208, 0.462) | 0.092  (-0.242, 0.427) | 0.110  Slight |
| 6.- Butter, margarine | <1 | 93.1 | 91.4 | 89.7 | 0.194  (-0.416, 0.805) | 0.346  (-0.150, 0.841) | 0.270  Fair |
| 7.- Sweet beverages | <1 | 93.1 | 80.2 | 77.8 | 0.065  (-0.433, 0.562) | 0.129  (-0.269, 0.526) | 0.097  Slight |
| 8.- Wine | 7 to14 | 6.9 | 5.2 | 94.8 | 0.545  (0.043, 1.046) | 0.545  (0.043, 1.046) | 0.545  Moderate |
| 9.- Legumes | ≥3 | 15.5 | 10.3 | 87.9 | 0.467  (0.097, 0.837) | 0.467  (0.097, 0.837) | 0.467  Moderate |
| 10.- Fish & seafood | ≥3 | 25.9 | 7.8 | 73.3 | 0.081  (-0.302, 0,464) | 0,114  (-0.272, 0.500) | 0.098  Slight |
| 11.- Desserts | <3 | 39.7 | 56.0 | 71.6 | 0.405  (0.176, 0.633) | 0.486  (0.263, 0.710) | 0.446  Moderate |
| 12.- Nuts | ≥3 | 17.2 | 27.6 | 74.1 | 0.316  (0.005, 0.628) | 0.219  (-0.107, 0.545) | 0.268  Fair |
| 13.- White over red meat^3^ | ≤1 or yes | 32.8 | 73.3 | 56.0 | 0.253  (0.033, 0.474) | 0.231  (0.012, 0.451) | 0.242  Fair |
| 14.- ‘Sofrito’ | ≥2 | 50.0 | 61.2 | 59.5 | 0.069  (-0.188, 0.326) | 0.310  (0.066, 0.555) | 0.190  Slight |
| Mean value |  | 40.8 | 49.0 | 77.7 |  |  |  |

^1^: Mean value of FFQ-MEDAS (1) and FFQ-MEDAS (2); ^2^: Not applicable (one of the variables is a constant when all answers scored the same value); ^3^: ≤1 for the 3d-FD and 'yes' for the FFQ-MEDAS; ^4^ к ≤ 0 no agreement (small negative values) or disagreement (large negative values), к = 0.01 − 0.20 slight, к = 0.21 − 0.40 fair, к = 0.41 − 0.60 moderate, к = 0.61 − 0.80 substantial, к = 0.81 – 1.0 almost perfect [26].
