# Supplementary material for: Exploring the Validity of the 14-Item Mediterranean Diet Adherence Screener (MEDAS): A Cross-National Study in Seven European Countries around the Mediterranean Region
Source: Nutrients. 2020 Sep 27;12(10):2960. doi: 10.3390/nu12102960 (PMC7601687; doi:10.3390/nu12102960)
Supplement: Supplementary file 1 [file nutrients-12-02960-s001.zip › Table S4.docx]

**Supplementary Table S4.-** Agreement between the FFQ-MEDAS and the 3d-FD: per-item validation analysis (κappa statistics) in the sample population from Portugal.

| Question | Score | 3d-FD  (% scoring 1) | FFQ-MEDAS^1^  (% scoring 1) | % Absolute agreement | κ (95%CI)  (3d-FD *vs* FFQ-MEDAS(1) | κ (95%CI)  (3d-FD *vs* FFQ-MEDAS(2) | κ (mean)  Level of agreement^3^ |
| --- | --- | --- | --- | --- | --- | --- | --- |
| 1.- Olive oil | yes | 88.4 | 97.7 | 88.4 | 0.133  (-0.372, 0.638) | 0.133  (-0.372, 0.638) | 0.133  Slight |
| 2.- Olive oil | ≥4 | 15.1 | 18.0 | 83.1 | 0.318  (0.017, 0.620) | 0.462  (0.193, 0.731) | 0.390  Fair |
| 3.- Vegetables | ≥2 | 11.6 | 42.4 | 66.9 | 0.280  (0.048, 0.511) | 0.224  (0.005, 0.442) | 0.252  Fair |
| 4.- Fresh fruits | ≥3 | 12.8 | 22.7 | 86.6 | 0.523  (0.272, 0.773) | 0.575  (0.340, 0.810) | 0.549  Moderate |
| 5.- Red & processed meat | <1 | 77.9 | 69.2 | 51.8 | -0.223  (-0.494, 0.047) | -0.233  (-0.499, 0.034) | -0.228  Disagreement |
| 6.- Butter, margarine | <1 | 77.9 | 57.6 | 59.9 | 0.143  (-0.082, 0.367) | 0.105  (-0.124, 0.333) | 0.124  Slight |
| 7.- Sweet beverages | <1 | 73.3 | 69.2 | 77.3 | 0.516  (0.309, 0.722) | 0.381  (0.158, 0.604) | 0.449  Moderate |
| 8.- Wine | 7 to14 | 11.6 | 5.2 | 87.8 | 0.178  (-0.253, 0.610) | 0.268  (-0.184, 0.721) | 0.223  Fair |
| 9.- Legumes | ≥3 | 9.3 | 20.4 | 77.3 | 0.117  (-0.222, 0.456) | 0.130  (-0.215, 0.475) | 0.124  Slight |
| 10.- Fish & seafood | ≥3 | 47.7 | 32.6 | 57.0 | 0.080  (-0.133, 0.294) | 0.171  (-0.041, 0.382) | 0.126  Slight |
| 11.- Desserts | <3 | 37.2 | 56.4 | 62.2 | 0.277  (0.079, 0.476) | 0.259  (0.061, 0.457) | 0.268  Fair |
| 12.- Nuts | ≥3 | 11.6 | 16.9 | 84.3 | 0.396  (0.093, 0.698) | 0.325  (0.002, 0.649) | 0.361  Fair |
| 13.- White over red meat^2^ | ≤1 or yes | 44.2 | 61.6 | 64.0 | 0.273  (0.074, 0.473) | 0.322  (0.127, 0.518) | 0.298  Fair |
| 14.- ‘Sofrito’ | ≥2 | 33.7 | 84.9 | 37.2 | -0.049  (-0.215, 0.118) | 0.002  (-0.165, 0.168) | -0.024  No agreement |
| Mean value |  | 39.5 | 46.8 | 70.2 |  |  |  |

^1^: Mean value of FFQ-MEDAS (1) and FFQ-MEDAS (2); ^2^: ≤1 for the 3d-FD and 'yes' for the FFQ-MEDAS. ^3^ к ≤ 0 no agreement (small negative values) or disagreement (large negative values), к = 0.01 − 0.20 slight, к = 0.21 − 0.40 fair, к = 0.41 − 0.60 moderate, к = 0.61 − 0.80 substantial, к = 0.81 – 1.0 almost perfect [26].
