# Supplementary material for: Exploring the Validity of the 14-Item Mediterranean Diet Adherence Screener (MEDAS): A Cross-National Study in Seven European Countries around the Mediterranean Region
Source: Nutrients. 2020 Sep 27;12(10):2960. doi: 10.3390/nu12102960 (PMC7601687; doi:10.3390/nu12102960)
Supplement: Supplementary file 1 [file nutrients-12-02960-s001.zip › Table S5.docx]

**Supplementary Table S5.-** Agreement between the FFQ-MEDAS and the 3d-FD: per-item validation analysis (κappa statistics) in the sample population from Cyprus.

| Question | Score | 3d-FD  (% scoring 1) | FFQ-MEDAS^1^  (% scoring 1) | % Absolute agreement | κ (95%CI)  (3d-FD *vs* FFQ-MEDAS(1) | κ (95%CI)  (3d-FD *vs* FFQ-MEDAS(2) | κ (mean)  Level of agreement^4^ |
| --- | --- | --- | --- | --- | --- | --- | --- |
| 1.- Olive oil | yes | 29.9 | 85.1 | 35.2 | -0.005  (-0.174, 0.165) | -0.001  (-0.165, 0.164) | -0.003  No agreement |
| 2.- Olive oil | ≥4 | 11.7 | 13.6 | 76.0 | -0.148  (-0.580, 0.285) | -0.020  (-0.448, 0.408) | -0.084  No agreement |
| 3.- Vegetables | ≥2 | 49.4 | 54.5 | 61.0 | 0.223  (0.006, 0.440) | 0.220  (0.002, 0.438) | 0.222  Fair |
| 4.- Fresh fruits | ≥3 | 16.9 | 26.0 | 79.2 | 0.352  (0.081, 0.624) | 0.429  (0.169, 0.688) | 0.391  Fair |
| 5.- Red & processed meat | <1 | 59.2 | 100.0 | 59.2 | NA^2^ | NA | NA |
| 6.- Butter, margarine | <1 | 80.5 | 50.0 | 50.0 | -0.190  (-0.375, -0.006) | 0.250  (0.008, 0.491) | 0.030  Slight |
| 7.- Sweet beverages | <1 | 98.7 | 55.9 | 55.9 | 0.031  (-0.211, 0.273) | -0.026  (-0.290, 0.237) | 0.003  No agreement |
| 8.- Wine | 7 to14 | 2.6 | 0.0 | 97.4 | NA | NA | NA |
| 9.- Legumes | ≥3 | 27.6 | 22.4 | 67.1 | 0.091  (-0.192, 0.374) | 0.161  (-0.117, 0.439) | 0.126  Slight |
| 10.- Fish & seafood | ≥3 | 33.8 | 1.3 | 67.5 | 0.099  (-0.200, 0.398) | NA | 0.099  Slight |
| 11.- Desserts | <3 | 54.5 | 89.0 | 55.2 | -0.041  (-0.283, 0.201) | 0.111  (-0.124, 0.347) | 0.035  Slight |
| 12.- Nuts | ≥3 | 15.6 | 28.6 | 75.3 | 0.263  (-0.015, 0.541) | 0.337  (0.069, 0.605) | 0.300  Fair |
| 13.- White over red meat^3^ | ≤1 or yes | 44.7 | 80.9 | 58.5 | 0.256  (0.049, 0.463) | 0.188  (-0.021, 0.396) | 0.222  Fair |
| 14.- ‘Sofrito’ | ≥2 | 25.0 | 31.6 | 61.6 | 0.055  (-0.199, 0.310) | 0.069  (-0.213, 0.351) | 0.062  Slight |
| Mean value |  | 39.3 | 45.6 | 64.2 |  |  |  |

^1^: Mean value of FFQ-MEDAS (1) and FFQ-MEDAS (2); ^2^: Not applicable (one of the variables is a constant when all answers scored the same value); ^3^: ≤1 for the 3d-FD and 'yes' for the FFQ-MEDAS; ^4^ к ≤ 0 no agreement (small negative values) or disagreement (large negative values), к = 0.01 − 0.20 slight, к = 0.21 − 0.40 fair, к = 0.41 − 0.60 moderate, к = 0.61 − 0.80 substantial, к = 0.81 – 1.0 almost perfect [26].
