# Supplementary material for: Exploring the Validity of the 14-Item Mediterranean Diet Adherence Screener (MEDAS): A Cross-National Study in Seven European Countries around the Mediterranean Region
Source: Nutrients. 2020 Sep 27;12(10):2960. doi: 10.3390/nu12102960 (PMC7601687; doi:10.3390/nu12102960)
Supplement: Supplementary file 1 [file nutrients-12-02960-s001.zip › Table S6.docx]

**Supplementary Table S6.-** Agreement between the FFQ-MEDAS and the 3d-FD: per-item validation analysis (κappa statistics) in the sample population from Greece.

| Question | Score | 3d-FD  (% scoring 1) | FFQ-MEDAS^1^  (% scoring 1) | % Absolute agreement | κ (95%CI)  (3d-FD *vs* FFQ-MEDAS(1) | κ (95%CI)  (3d-FD *vs* FFQ-MEDAS(2) | κ (mean)  Level of agreement^4^ |
| --- | --- | --- | --- | --- | --- | --- | --- |
| 1.- Olive oil | yes | 98.0 | 100.0 | 98.0 | NA^2^ | NA | NA |
| 2.- Olive oil | ≥4 | 68.0 | 48.0 | 74.0 | 0.495  (0.258, 0.731) | 0.480  (0.237, 0.723) | 0.488  Moderate |
| 3.- Vegetables | ≥2 | 54.0 | 69.0 | 75.0 | 0.386  (0.126, 0.646) | 0.584  (0.353, 0.815) | 0.485  Moderate |
| 4.- Fresh fruits | ≥3 | 58.0 | 50.0 | 78.0 | 0.520  (0.283, 0.757) | 0.600  (0.378, 0.822) | 0.560  Moderate |
| 5.- Red & processed meat | <1 | 26.0 | 39.0 | 75.0 | 0.480  (0.224, 0.737) | 0.399  (0.118, 0.680) | 0.440  Moderate |
| 6.- Butter, margarine | <1 | 18.0 | 27.0 | 81.0 | 0.377  (0.031, 0.722) | 0.532  (0.255, 0.809) | 0.455  Moderate |
| 7.- Sweet beverages | <1 | 32.0 | 40.0 | 68.0 | 0.382  (0.121, 0.644) | 0.231  (-0.065, 0.528) | 0.307  Fair |
| 8.- Wine | 7 to14 | 28.0 | 24.0 | 66.0 | -0.008  (-0.366, 0.349) | 0.239  (-0.083, 0.561) | 0.116  Slight |
| 9.- Legumes | ≥3 | 42.0 | 36.0 | 78.0 | 0.501  (0.255, 0.747) | 0.578  (0.345, 0.812) | 0.540  Moderate |
| 10.- Fish & seafood | ≥3 | 24.0 | 15.0 | 79.0 | 0.339  (-0.028, 0.705) | 0.341  (-0.004, 0.685) | 0.340  Fair |
| 11.- Desserts | <3 | 10.0 | 16.0 | 78.0 | 0.035  (-0.469, 0.539) | 0.035  (-0.469, 0.539) | 0.035  Poor |
| 12.- Nuts | ≥3 | 22.0 | 26.0 | 94.0 | 0.841  (0.666, 1.015) | 0.831  (0.645, 1.016) | 0.836  Very good |
| 13.- White over red meat^3^ | ≤1 or yes | 98.0 | 95.0 | 97.0 | 1.000  (1.000, 1.000) | 0.380  (-0.300, 1.060) | 0.690  Good |
| 14.- ‘Sofrito’ | ≥2 | 42.0 | 44.0 | 96.0 | 0.879  (0.747, 1.012) | 0.959  (0.878, 1.039) | 0.919  Very good |
| Mean value |  | 44.3 | 44.9 | 81.2 |  |  |  |

^1^: Mean value of FFQ-MEDAS (1) and FFQ-MEDAS (2); ^2^: Not applicable (one of the variables is a constant when all answers scored the same value); ^3^: ≤1 for the 3d-FD and 'yes' for the FFQ-MEDAS; ^4^ к ≤ 0 no agreement (small negative values) or disagreement (large negative values), к = 0.01 − 0.20 slight, к = 0.21 − 0.40 fair, к = 0.41 − 0.60 moderate, к = 0.61 − 0.80 substantial, к = 0.81 – 1.0 almost perfect [26].
