# Supplementary material for: Exploring the Validity of the 14-Item Mediterranean Diet Adherence Screener (MEDAS): A Cross-National Study in Seven European Countries around the Mediterranean Region
Source: Nutrients. 2020 Sep 27;12(10):2960. doi: 10.3390/nu12102960 (PMC7601687; doi:10.3390/nu12102960)
Supplement: Supplementary file 1 [file nutrients-12-02960-s001.zip › Table S7.docx]

**Supplementary Table S7.-** Agreement between the FFQ-MEDAS and the 3d-FD: per- item validation analysis (κappa statistics) in the sample population from Macedonia.

| Question | Score | 3d-FD  (% scoring 1) | FFQ-MEDAS^1^  (% scoring 1) | % Absolute agreement | κ (95%CI)  (3d-FD *vs* FFQ-MEDAS(1) | κ (95%CI)  (3d-FD *vs* FFQ-MEDAS(2) | κ (mean)  Level of agreement^4^ |
| --- | --- | --- | --- | --- | --- | --- | --- |
| 1.- Olive oil | yes | 2.3 | 5.8 | 94.2 | 0.482  (-0.289, 1.183) | -0.032  (-1.158, 1.094) | 0.225  Fair |
| 2.- Olive oil | ≥4 | 0.0 | 2.3 | 97.7 | NA^2^ | NA | NA |
| 3.- Vegetables | ≥2 | 44.2 | 28.0 | 60.5 | 0.220  (-0.083, 0.523) | 0.112  (-0.200, 0.425) | 0.166  Slight |
| 4.- Fresh fruits | ≥3 | 14.0 | 2.3 | 83.7 | -0.042  (-0.748, 0.664) | -0.042  (-0.748, 0.664) | -0.042  No agreement |
| 5.- Red & processed meat | <1 | 81.4 | 91.9 | 89.5 | 0.494  (0.077, 0.911) | 0.619  (0.264, 0.975) | 0.557  Moderate |
| 6.- Butter, margarine | <1 | 72.1 | 68.6 | 43.4 | -0.272  (-0.643, 0.100) | -0.342  (-0.682, -0.002) | -0.307  Disagreement |
| 7.- Sweet beverages | <1 | 79.1 | 67.4 | 65.1 | 0.125  (-0.233, 0.482) | 0.125  (-0.233, 0.482) | 0.125  Slight |
| 8.- Wine | 7 to14 | 7.0 | 2.3 | 95.4 | 0.482  (-0.219, 1,183) | 0.482  (-0.219, 1,183) | 0.482  Moderate |
| 9.- Legumes | ≥3 | 7.0 | 20.9 | 72.1 | -0.123  (-0.606, 0.360) | -0.108  (-0.710, 0.494) | -0.116  Disagreement |
| 10.- Fish & seafood | ≥3 | 11.6 | 1.2 | 86.1 | NA | -0.040  (-0.812, 0.732) | -0.040  No agreement |
| 11.- Desserts | <3 | 23.3 | 45.4 | 57.0 | 0.058  (-0.259, 0.374) | 0.130  (-0.176, 0.437) | 0.094  Slight |
| 12.- Nuts | ≥3 | 20.9 | 23.3 | 67.4 | -0.013  (-0.426, 0.401) | 0.122  (-0.276, 0.521) | 0.055  Slight |
| 13.- White over red meat^3^ | ≤1 or yes | 65.1 | 45.4 | 52.3 | 0.089  (-0.203, 0.381) | 0.056  (-0.232, 0.345) | 0.073  Slight |
| 14.- ‘Sofrito’ | ≥2 | 41.9 | 75.6 | 57.0 | 0.187  (-0.086, 0.460) | 0.224  (-0.049, 0.498) | 0.206  Fair |
| Mean value |  | 33.6 | 34.3 | 73.0 |  |  |  |

^1^: Mean value of FFQ-MEDAS (1) and FFQ-MEDAS (2); ^2^: Not applicable (one of the variables is a constant when all answers scored the same value); ^3^: ≤1 for the 3d-FD and 'yes' for the FFQ-MEDAS; ^4^ к ≤ 0 no agreement (small negative values) or disagreement (large negative values), к = 0.01 − 0.20 slight, к = 0.21 − 0.40 fair, к = 0.41 − 0.60 moderate, к = 0.61 − 0.80 substantial, к = 0.81 – 1.0 almost perfect [26].
