# Supplementary material for: Exploring the Validity of the 14-Item Mediterranean Diet Adherence Screener (MEDAS): A Cross-National Study in Seven European Countries around the Mediterranean Region
Source: Nutrients. 2020 Sep 27;12(10):2960. doi: 10.3390/nu12102960 (PMC7601687; doi:10.3390/nu12102960)
Supplement: Supplementary file 1 [file nutrients-12-02960-s001.zip › Table S8.docx]

**Supplementary Table S8.-** Agreement between the FFQ-MEDAS and the 3d-FD: per- item validation analysis (κappa statistics) in the sample population from Bulgaria.

| Question | Score | 3d-FD  (% scoring 1) | FFQ-MEDAS^1^  (% scoring 1) | % Absolute agreement | κ (95%CI)  (3d-FD *vs* FFQ-MEDAS(1) | κ (95%CI)  (3d-FD *vs* FFQ-MEDAS(2) | κ (mean)  Level of agreement^4^ |
| --- | --- | --- | --- | --- | --- | --- | --- |
| 1.- Olive oil | yes | 27.1 | 27.1 | 94.9 | 0.914  (0.797, 1.031) | 0.828  (0.666, 0.991) | 0.871  Very good |
| 2.- Olive oil | ≥4 | 1.7 | 1.7 | 96.6 | -0.017  (-1.403, 1.368) | -0.017  (-1.403, 1.368) | -0.017  No agreement |
| 3.- Vegetables | ≥2 | 11.9 | 100.0 | 11.9 | NA^2^ | NA | NA |
| 4.- Fresh fruits | ≥3 | 5.1 | 0.0 | 94.9 | NA | NA | NA |
| 5.- Red & processed meat | <1 | 71.2 | 0.0 | 28.8 | NA | NA | NA |
| 6.- Butter, margarine | <1 | 86.4 | 58.5 | 63.6 | 0.199  (-0.076, 0.474) | 0.137  (-0.149, 0.423) | 0.168  Slight |
| 7.- Sweet beverages | <1 | 94.9 | 66.1 | 69.5 | 0.177  (-0.140, 0.494) | 0.103  (-0.242, 0.448) | 0.140  Slight |
| 8.- Wine | 7 to14 | 11.9 | 11.9 | 93.2 | 0.676  (0.369, 0.983) | 0.676  (0.369, 0.983) | 0.676  Good |
| 9.- Legumes | ≥3 | 15.3 | 0.0 | 84.8 | NA | NA | NA |
| 10.- Fish & seafood | ≥3 | 5.1 | 0.0 | 94.9 | NA | NA | NA |
| 11.- Desserts | <3 | 62.7 | 100.0 | 62.7 | NA | NA | NA |
| 12.- Nuts | ≥3 | 11.9 | 0.0 | 88.1 | NA | NA | NA |
| 13.- White over red meat^3^ | ≤1 or yes | 22.0 | 48.3 | 53.4 | 0.042  (-0.216, 0.299) | 0.058  (-0.204, 0.320) | 0.050  Slight |
| 14.- ‘Sofrito’ | ≥2 | 0.0 | 33.9 | 66.1 | NA | NA | NA |
| Mean value |  | 30.5 | 32.0 | 71.7 |  |  |  |

^1^: Mean value of FFQ-MEDAS (1) and FFQ-MEDAS (2); ^2^: Not applicable (one of the variables is a constant when all answers scored the same value); ^3^: ≤1 for the 3d-FD and 'yes' for the FFQ-MEDAS; ^4^ к ≤ 0 no agreement (small negative values) or disagreement (large negative values), к = 0.01 − 0.20 slight, к = 0.21 − 0.40 fair, к = 0.41 − 0.60 moderate, к = 0.61 − 0.80 substantial, к = 0.81 – 1.0 almost perfect [26].
